# Lower bile duct metastasis from rectal cancer after surgery for liver metastasis and intrahepatic bile duct metastasis: a case report

**DOI:** 10.1186/s12893-020-00799-4

**Published:** 2020-06-17

**Authors:** Yoichi Nakagawa, Atsuyuki Maeda, Kazuaki Seita, Yuji Kaneoka

**Affiliations:** 1 Department of Pediatric Surgery, Ogaki Municipal Hospital, 4-86, Minaminokawa-cho, Ogaki City, Gifu Pref 503-8502 Japan; 2 Department of Surgery, Ogaki Municipal Hospital, 4-86, Minaminokawa-cho, Ogaki City, Gifu Pref 503-8502 Japan

**Keywords:** Lower bile duct, Metastasis, Intrabiliary growth, Liver metastasis, Cholangiocarcinoma

## Abstract

**Background:**

Biliary metastasis of colorectal cancer is a manifestation of metastatic liver carcinoma, and is often difficult to differentiate from cholangiocarcinoma. Further, lower bile duct metastasis of colorectal cancer is rare. We report the case of a 74-year-old woman who underwent pylorus-preserving pancreatoduodenectomy for lower bile duct metastasis of rectal cancer.

**Case presentation:**

The patient had undergone laparoscopic low anterior resection for rectal cancer (pT3N0M0 stage IIA) 6 years ago, laparoscopic anterior liver resection for liver metastasis (Couinaud segment V) 3 years ago, and left and caudal lobectomy with extrahepatic bile duct resection for left intrahepatic bile duct metastasis 6 months ago. A follow-up examination showed a 15 mm mass in the common bile duct, for which she underwent pylorus-preserving pancreatoduodenectomy. Histological and immunohistological examination of the specimens revealed similar cytokeratin (CK) expression patterns, which were negative for CK7 and positive for CK20. Therefore, the definitive diagnosis was metastasis from rectal cancer.

**Conclusions:**

In summary, we encountered a case of lower bile duct metastasis from rectal cancer, which is often difficult to differentiate from cholangiocarcinoma. In such patients, CK7 and CK20 expression patterns are important in differentiating the two. The mechanism of metastasis in this case was considered to be through cancer cell implantation from lymphatic spread, or through distant metastasis of the primary cancer.

## Background

Biliary metastasis of colorectal cancer is a manifestation of metastatic liver carcinoma [1]. Intrabiliary growth of liver metastasis of colon cancer has been reported not to be aggressive and to have a good prognosis. In terms of incidence, this metastasis comprises approximately 10% of colorectal liver metastases [2, 3]. A few cases of lower bile duct metastasis from colorectal cancer have been reported. Here, we report a case of lower bile duct metastasis from rectal cancer for which pylorus-preserving pancreatoduodenectomy was performed. The patient had previously undergone laparoscopic low anterior resection, laparoscopic anterior liver resection, and left and caudal lobectomy with extrahepatic bile duct resection.

## Case presentation

**Fig. 1:**
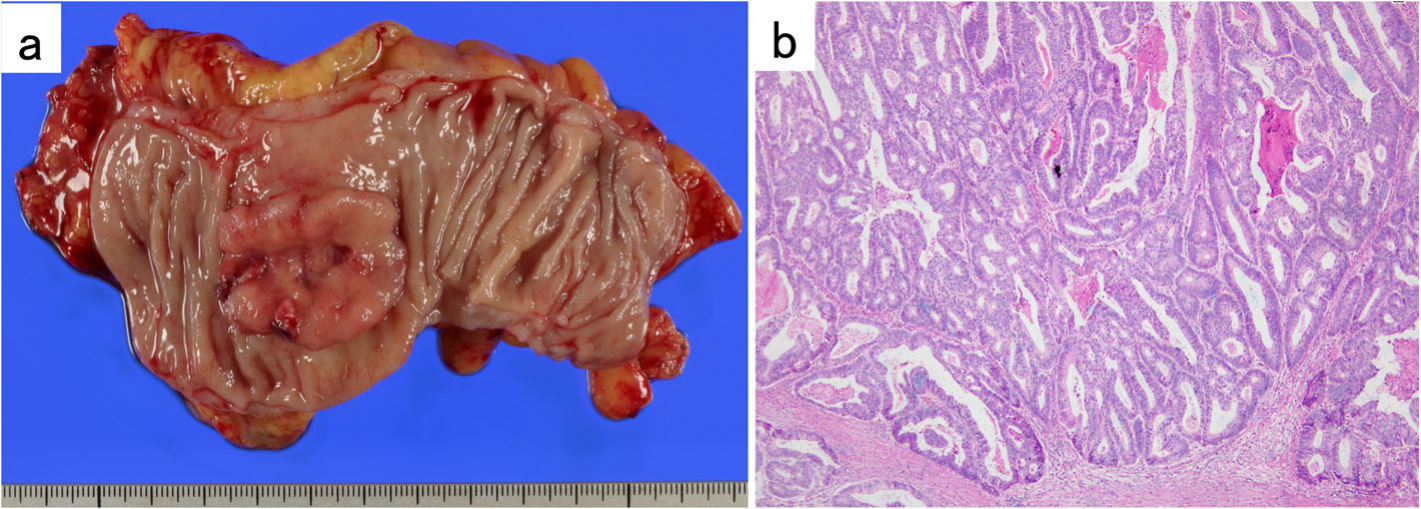
**a** Macroscopic findings of the resected specimen. **b** Hematoxylin-Eosin stained specimen (× 40, original magnification). Histological examination revealed a well differentiated tubular adenocarcinoma

A 68-year-old woman, who had a history of hypertension, hyperlipidemia, and diabetes mellitus, underwent laparoscopic low anterior resection (LLAR) for treatment of her rectal cancer in December 2011. Histological examination revealed a well differentiated tubular adenocarcinoma, pT3N0M0 stage II A according to the Union for International Cancer Control (UICC) TNM classification system (Fig. 1). The surgical margin was negative, and there was no vascular or lymphatic invasion. No adjuvant therapy was given, and there was no evidence of recurrence for 3 years.

**Fig. 2:**
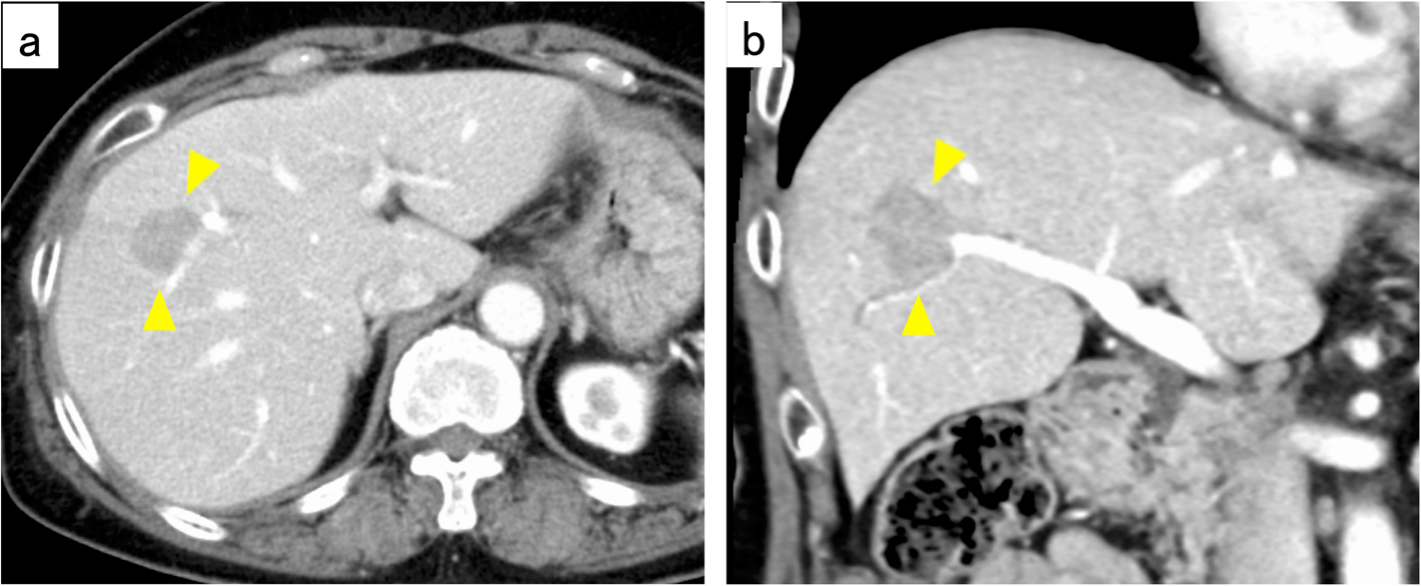
Contrast-enhanced CT scan revealed a tumor measuring 2.5 cm in diameter at Couinaud segment V of the liver (**a** Axial, **b** Coronal)

**Fig. 3:**
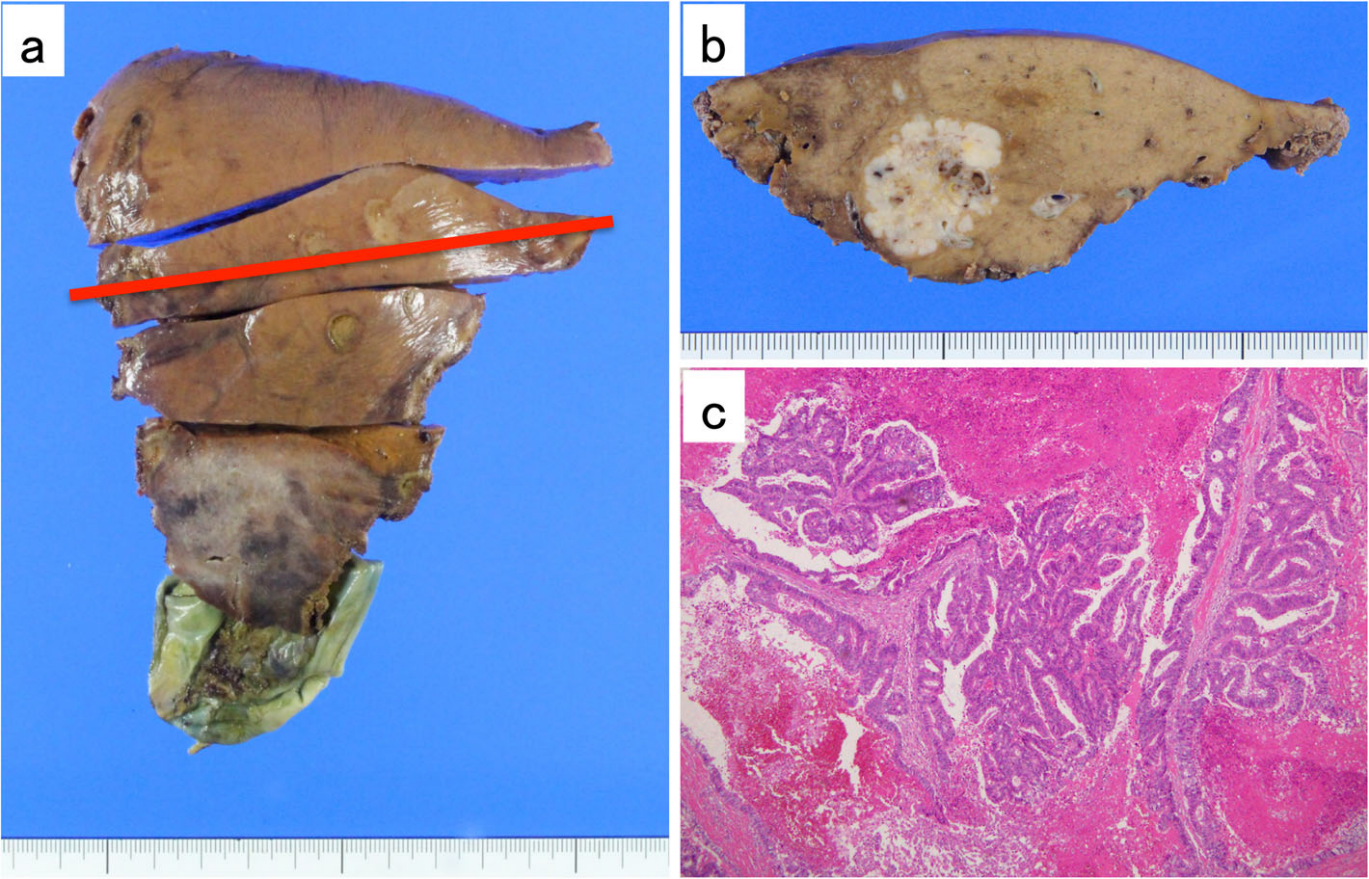
**a**, **b** Macroscopic findings of the resected specimen. **c** Hematoxylin-Eosin stained specimen (× 40, original magnification). Histological examination revealed a well- to moderately differentiated adenocarcinoma forming a tubular structure

Three years after the LLAR, the follow-up computed tomography (CT) scan revealed liver metastasis in Couinaud segment V (Fig. 2). A diagnosis of liver metastasis of rectal cancer was made, and she therefore underwent a laparoscopic anterior segment liver resection in February 2015. Histological examination revealed a moderately differentiated tubular adenocarcinoma without vascular or bile duct invasion, and with a preserved surgical margin (Fig. 3).

**Fig. 4:**
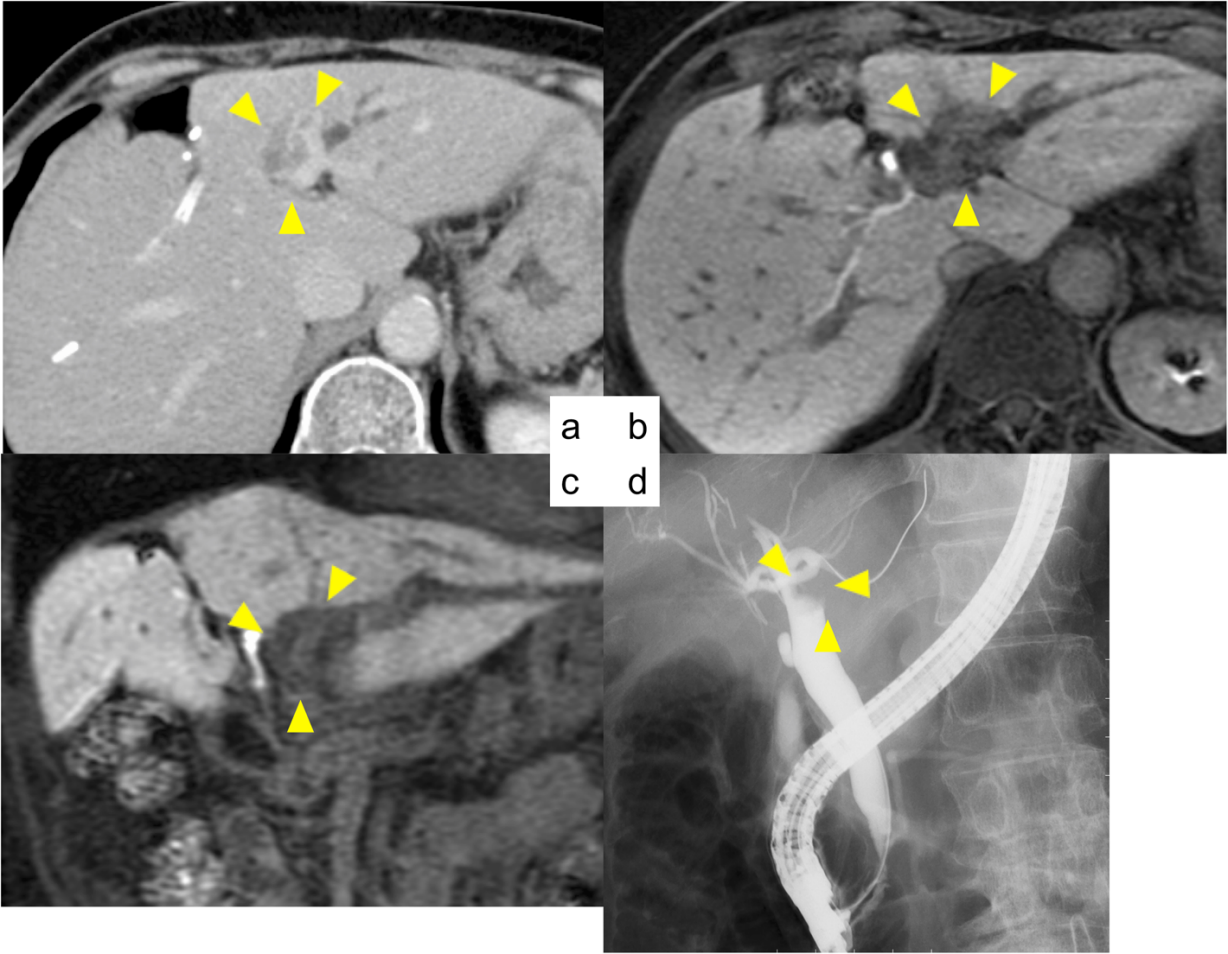
**a** Contrast-enhanced CT scan revealed dilation of left intrahepatic bile duct (8 mm) and a high-density mass in segments II and III. **b**, **c** MRCP also revealed a 20 × 14 mm mass in segments II and III. **d** ERCP revealed a shadow defect in segments II and III. No other tumor or defect was detected

**Fig. 5:**
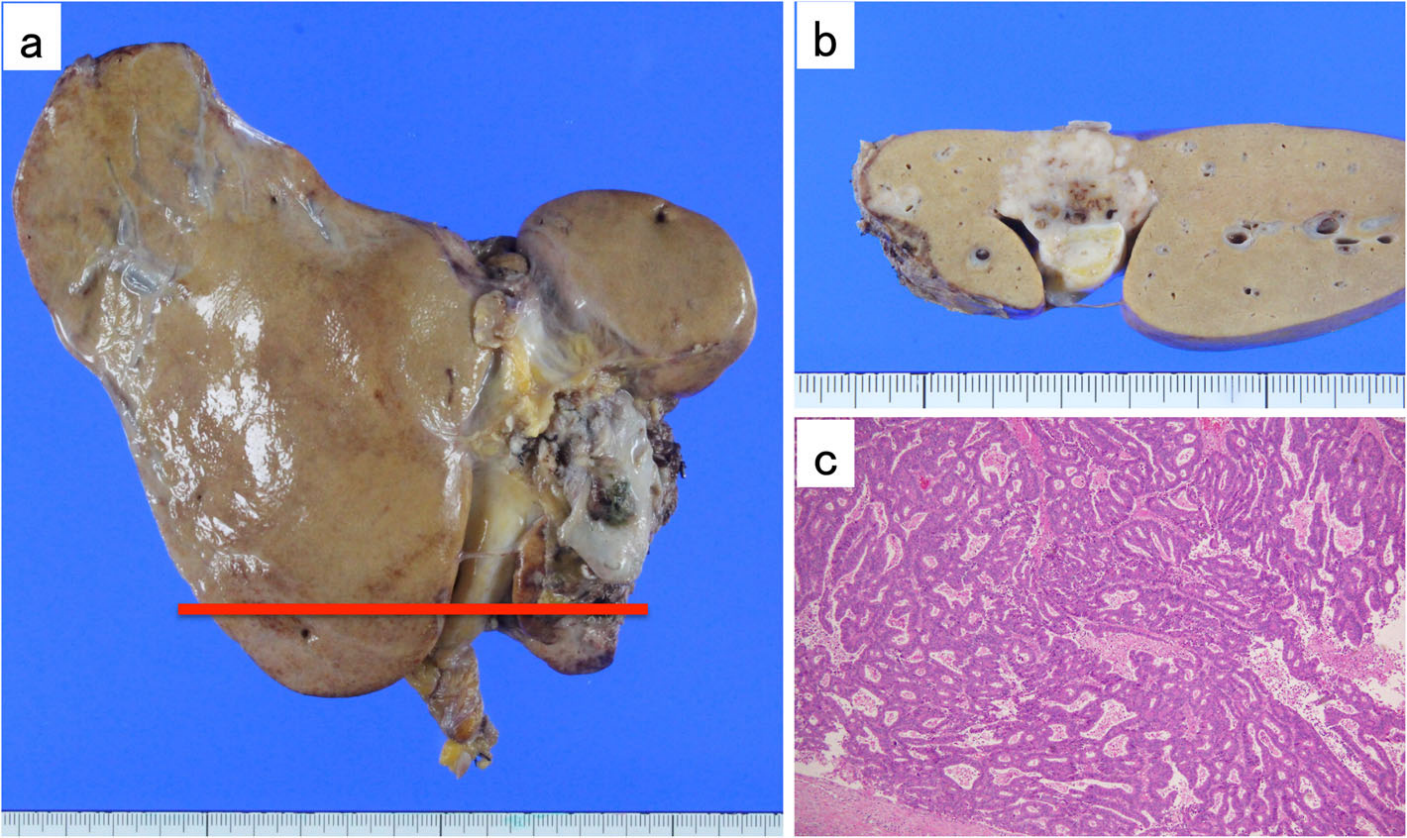
**a**, **b** Macroscopic findings of the resected specimen. **c** Hematoxylin-Eosin stained specimen (× 40, original magnification). Histological examination revealed a moderately differentiated tubular adenocarcinoma in segments II and III that invaded to the left hepatic duct and liver parenchyma forming a 20 mm nodule

Two years after undergoing hepatectomy, the follow-up CT scan revealed dilation of the left intrahepatic bile duct and a high-density mass in the common trunk of segment II and segment III (B23). Magnetic resonance cholangiopancreatography (MRCP) and endoscopic retrograde cholangiopancreatography (ERCP) also revealed a mass in B23 (Fig. 4). The biopsy findings from ERCP confirmed adenocarcinoma. Therefore, she underwent left and caudal lobectomy with extrahepatic bile duct resection in April 2017. Histological examination revealed a moderately differentiated tubular adenocarcinoma in segments II and III that invaded into the left hepatic duct and the liver parenchyma, forming a 20 mm nodule. There was no vascular invasion, and the surgical margin was preserved (Fig. 5). The postoperative diagnosis was intrahepatic cholangiocarcinoma, pT2N0M0 stage II according to the American Joint Committee on Cancer (AJCC) 8th edition.

**Fig. 6:**
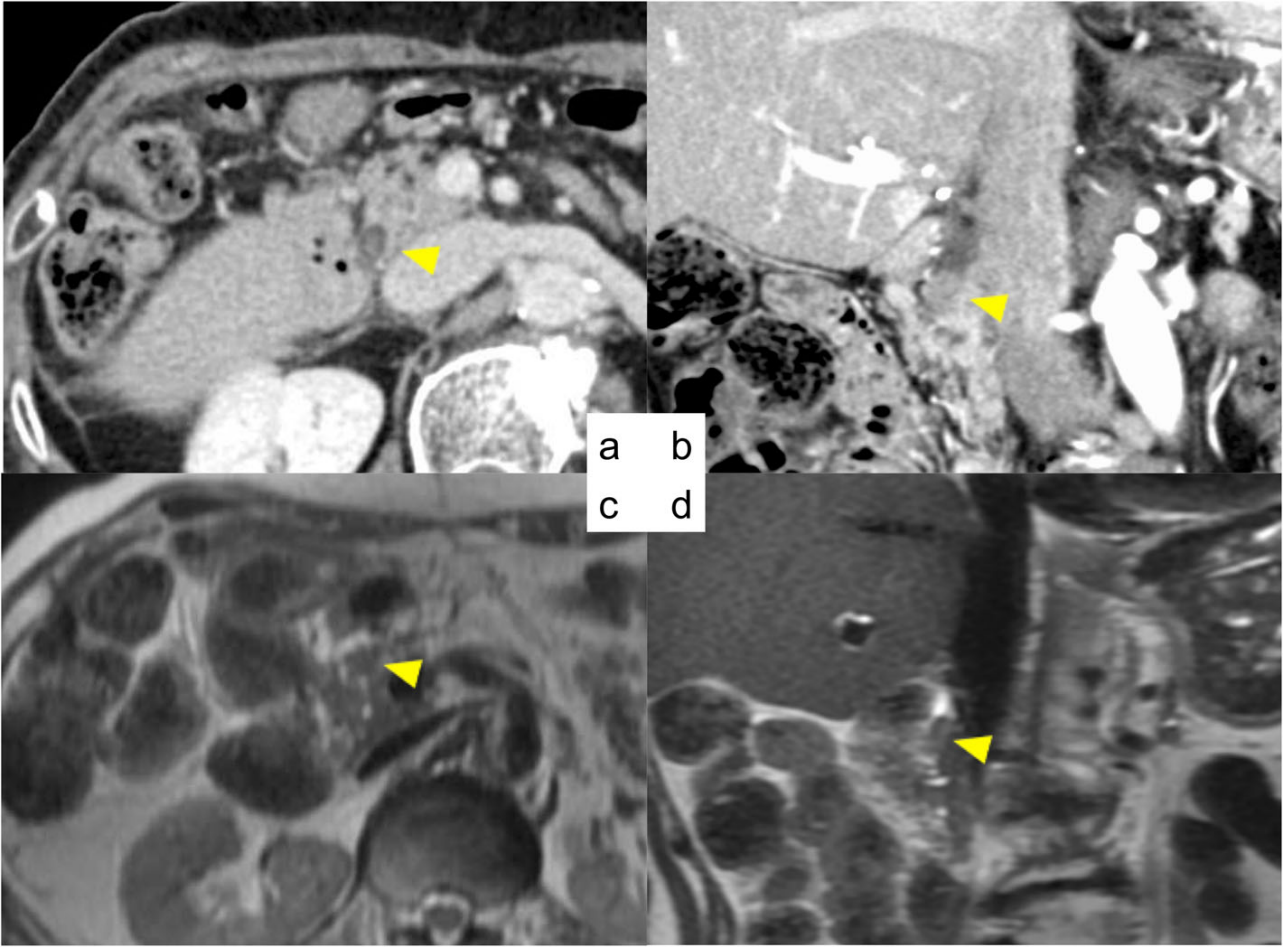
**a**, **b** Contrast enhanced CT scan revealed a 15 mm mass with enhancement in the early phase at the common bile duct. **c**, **d** MRCP revealed a 15 × 6 mm low intensity mass at the end of the common bile duct

**Fig. 7:**
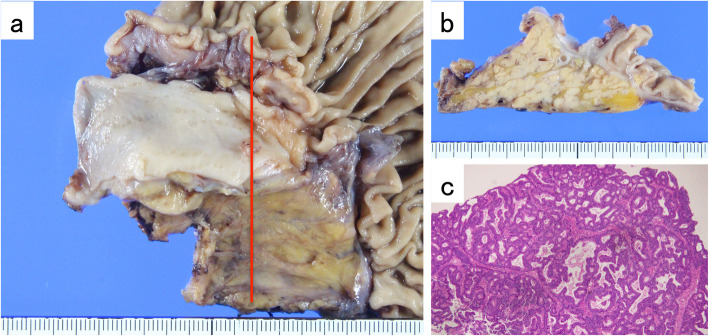
**a**, **b** Macroscopic findings of the resected specimen. **c** Hematoxylin-Eosin stained specimen (× 40, original magnification). Histological examination revealed a well to moderately differentiated tubular adenocarcinoma with LN 8a metastasis

In October 2017, a follow-up CT scan revealed a 15 mm mass with enhancement in the early phase in the common bile duct, and lymphadenopathy at 8a. MRCP also revealed a 15 × 6 mm low intensity mass at the end of the common bile duct, and a slightly high diffusion-weighted imaging (DWI) but no corresponding low signal on the apparent diffusion coefficient (ADC) map (Fig. 6). A diagnosis of lower bile duct cancer was made. Consequently, she received pylorus-preserving pancreatoduodenectomy in November 2017. Histological examination revealed a well to moderately differentiated tubular adenocarcinoma with preserved surgical margin. Although there was no vascular invasion, lymph node (LN) 8a metastasis (2/2) was observed (Fig. 7).

**Fig. 8:**
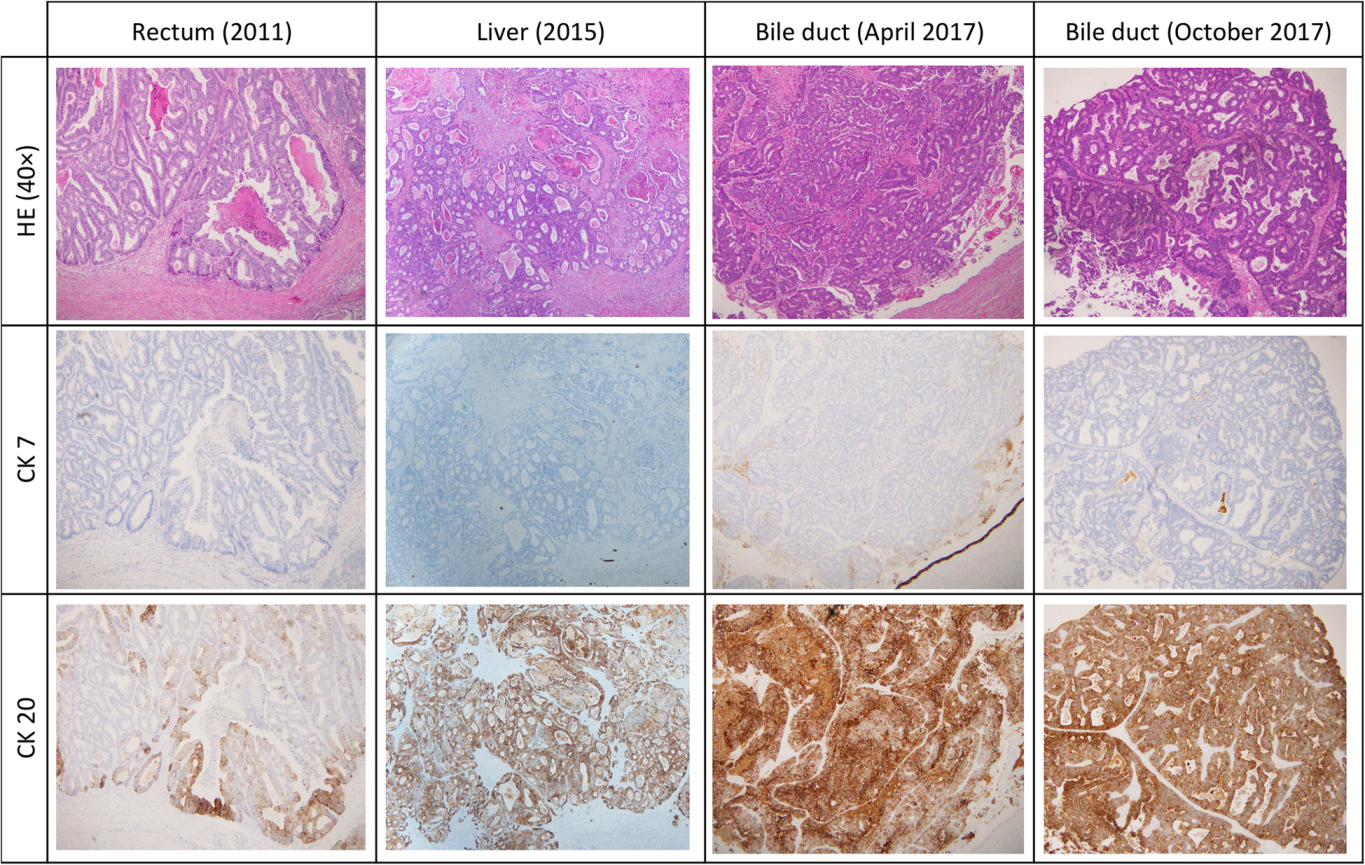
Immunohistochemical staining of all specimens in this case (× 40, original magnification). All specimens revealed a well to moderately differentiated tubular adenocarcinoma. They also revealed similar CK7 and CK20 expression patterns: negative for CK7 and positive for CK20

Upon comparison, the histology of the current surgical specimen was found to be similar to those from the earlier surgeries. Immunological findings also revealed similar cytokeratin (CK) expression patterns of the specimens, which were negative for CK7 and positive for CK20 (Fig. 8), indicating that the tumor originated from the colon or rectum. Based on these findings, a definitive diagnosis of rectal cancer with metastases to the liver, intrahepatic bile duct, and lower bile duct was made. The patient was followed up as an outpatient for 2 years, and no apparent recurrence or metastasis was observed.

## Discussion and conclusion

Biliary metastasis of colorectal cancer is quite common and has a good prognosis. A characteristic imaging finding for biliary metastasis is dilation of the intrahepatic bile ducts, and this often makes it difficult to differentiate intrahepatic cholangiocarcinoma from metastatic liver carcinoma. Immunohistological staining is useful for this differentiation. CK7 negative and CK20 positive expression pattern has a 93% positive predictive value for colorectal metastatic cancer. The pattern in our patient had only a 4% positive predictive value for cholangiocarcinoma [4].

In this case, based on the immunohistological findings, we determined that the intrahepatic bile duct cancer in April 2017 and the lower bile duct cancer in October 2017 were metastases of the rectal cancer in 2011. Only a few cases of lower bile duct metastasis from colorectal cancer have been reported [5–7]. Sano et al. suggested that the mechanism of metastasis was through cancer cell implantation. This may possibly be due to spontaneous shedding of cancer cells, or may arise from a complication of percutaneous transhepatic biliary drainage [6]. The rate of percutaneous transhepatic biliary drainage catheter tract recurrence has been shown to be significantly high in cases of well differentiated papillary cholangiocarcinoma, because it is thought to be fragile and susceptible to collapse [7]. Kawakatsu et al. reported that the mechanism of metastasis involves cancer cell implantation rather than hematogenous lower bile duct metastasis, due to this fragile nature of well differentiated papillary adenocarcinoma [8]. If this theory is correct, because metastasis is a local disease, the cancer may be curable by complete resection.

As mentioned above, our case likely followed the same process of metastasis. Considering the rarity of bile duct metastasis of colorectal cancer, it is quite unlikely that bile duct metastasis occurred twice in this case. Our histological examination revealed that the surgical margin was preserved and that there was no vascular, lymphatic, or neuronal invasion. We considered that the mechanism of metastasis involved cancer cell implantation. Implantation was considered to occur at the injured bile duct during the ERCP, or at the site of the resection stump during the left and caudal lobectomy with extrahepatic bile duct resection. However, it is uncertain whether seeding by biopsy was involved.

Our case showed some differences from former reports. There was isolated LN 8a metastasis in our case. Considering the mechanism of LN metastasis, there are two possibilities: hematogenous metastasis from primary rectal cancer or lymphatic invasion from former metastases. If hematogenous metastasis is the cause, the metastasis is not a local disease, but a systemic one; hence, chemotherapy may be needed in some cases. In contrast, if lymphatic invasion is the cause, the metastasis is a local disease and may need complete excision. In this case, we considered the mechanism of LN metastasis to be lymphatic invasion from either left intrahepatic bile duct metastasis of rectal cancer or lower bile duct metastasis of rectal cancer. In general, adjuvant chemotherapy after hepatectomy for bile duct metastasis of colorectal cancer, or even liver metastasis of colorectal cancer, does not have sufficient evidence of prognostic improvement. Our institution’s policy, therefore, is not to perform adjuvant chemotherapy after complete resection for distant metastasis.

We have been following up on the patient for over 2 years without chemotherapy, and there has been no sign of metastasis and recurrence. If any evidence occurs, she may be considered for chemotherapy. Further studies in more patients are needed to evaluate whether chemotherapy will prevent recurrence or not.

In conclusion, we managed a case of lower bile duct metastasis of rectal cancer in which CK7 and CK20 expression patterns were useful to confirm whether it was liver metastasis or cholangiocarcinoma. The mechanism of metastasis was considered to be either cancer cell implantation with lymphatic invasion or distal metastasis of the primary cancer.

## Data Availability

The datasets during the current study are available from the corresponding author on reasonable request.

## Abbreviations

LLAR: Laparoscopic low anterior resection
UICC: Union for International Cancer Control
CT: Computed tomography
MRCP: Magnetic resonance cholangiopancreatography
ERCP: Endoscopic retrograde cholangiopancreatography
DWI: Diffusion-weighted imaging
ADC: Apparent diffusion coefficient
LN: Lymph node
CK: Cytokeratin
